# Distinct roles for complement in glomerulonephritis and atherosclerosis revealed in mice with a combination of lupus and hyperlipidemia

**DOI:** 10.1002/art.34451

**Published:** 2012-08

**Authors:** Myles J Lewis, Talat H Malik, Liliane Fossati-Jimack, Daniele Carassiti, H Terence Cook, Dorian O Haskard, Marina Botto

**Affiliations:** Imperial College LondonLondon, UK

## Abstract

*Objective* Although the accelerating effect of systemic lupus erythematosus (SLE) on atherosclerosis is well established, the underlying mechanisms are unknown. The aim of this study was to explore the hypothesis that lupus autoimmunity modulates the effect of hypercholesterolemia in driving arterial pathologic development.

>*Methods* Low-density lipoprotein receptor–deficient (*Ldlr*^−/−^) mice were crossed with B6.129-*Sle16* (*Sle16*)–congenic autoimmune mice to obtain *Sle16. Ldlr*^−/−^ mice, which were compared with *Ldlr*^−/−^ and *Sle16* control mice. All mice were fed either a low-fat or high-fat diet. Groups of mice were compared, by strain and by diet group, for features of accelerated atherosclerosis and autoimmunity.

*Results* Presence of the *Sle16* locus significantly increased the extent of atherosclerosis in *Ldlr*^−/−^ mice. Circulating C3 levels were significantly reduced in *Sle16.Ldlr*^−/−^ mice compared to *Ldlr*^−/−^ control mice and this was paralleled by a marked reduction in arterial lesion C3 deposition despite similar levels of IgG deposition between the groups. Increased numbers of apoptotic cells in plaques were observed in the high-fat–fed *Sle16.Ldlr*^−/−^ mice, consistent with the observed defective clearance of cellular debris. After receiving the high-fat diet, *Sle16.Ldlr*^−/−^ mice developed glomerulonephritis and displayed enhanced glomerular C3 deposition.

*Conclusion* These results indicate that accelerated atherosclerosis and renal inflammation in SLE are closely linked via immune complex formation and systemic complement depletion. However, whereas hyperlipidemia will enhance renal immune complex–mediated complement activation and the development of nephritis, accelerated atherosclerosis is, instead, related to complement depletion and a reduction in the uptake of apoptotic/necrotic debris. These results suggest that aggressive treatment of hyperlipidemia in patients with SLE may reduce the occurrence of lupus nephritis, as well as diminish the risk of accelerated atherosclerosis.

The association between systemic lupus erythematosus (SLE) and cardiovascular events is well established in epidemiologic studies. In particular, results have shown a substantial increase in angina and myocardial infarction in patients with SLE (1). Imaging studies have shown an increased prevalence of carotid plaque and coronary calcification in patients with SLE, suggesting that there is an accelerated burden of atherosclerosis in these patients (2, 3). Given the inflammatory nature of atherosclerosis, the acceleration of disease in the presence of SLE is perhaps not surprising. However, the exact underlying mechanisms involved are still poorly defined.

Studies utilizing murine models of atherosclerosis in combination with a lupus-like disease have been reported (4–8). Accelerated atherosclerosis has been described in apolipoprotein E (ApoE)–deficient mice with either the *gld* or *lpr* mutation (4, 6, 8), and in bone marrow chimeras of *gld* mice transplanted into low-density lipoprotein (LDL) receptor–deficient (*Ldlr*^−/−^) mice (*gld→Ldlr*^−/−^) (7). Radiation chimeras using the triple-congenic lupus mouse strain B6.*Sle1.2.3* transplanted into *Ldlr*^−/−^ mice (*Sle1.2.3→Ldlr*^−/−^) also showed increased atherosclerosis, which was thought to be due to systemic activation of B and T cells (5).

As immune complex (IC) formation is a feature of SLE, it seems logical to suppose that accelerated atherosclerosis in SLE could be attributed to increased IC deposition within the arterial walls, with subsequent activation of complement. However, there is no experimental evidence to support this hypothesis. Hypocomplementemia has been linked to defective clearance of apoptotic cells (9), which is considered to be a key pathogenetic mechanism in the development of both lupus and atherosclerosis (4). Acceleration of murine atherosclerosis attributed to accumulation of apoptotic cells within atherosclerotic lesions has been described in the context of deficiency of complement C1q (10), Mer receptor tyrosine kinase (11, 12), and lactadherin (13). A lupus-like phenotype associated with defective uptake of apoptotic cells has also been demonstrated in these mice (9, 14, 15). Even though all these findings indicate that synergistic mechanisms may operate in both conditions, whether the same pathways lead to an accelerated development of atherosclerosis and increased renal damage remains to be elucidated.

Herein we describe a novel mouse model of lupus and atherosclerosis (*Sle16.Ldlr*^−/−^) generated by crossing *Ldlr*^−/−^ mice with the congenic line *Sle16* (16). The *Sle16* line carries, on chromosome 1, an interval originating in the 129 mouse strain, and these mice develop autoantibodies and mild renal inflammation with IC deposition without proteinuria (16). We observed that both nephritis and atherosclerosis were accelerated in the *Sle16.Ldlr*^−/−^ mice, suggesting that synergistic interactions take place between systemic immune dysregulation and dyslipidemia. Importantly, our data indicate that distinct complement-related mechanisms operate in the kidney when compared to the arterial walls.

## MATERIALS AND METHODS

### Mice

*Sle16* mice, previously known as B6.129chr1b (16), were crossed with *Ldlr*^−/−^ mice on a C57BL/6 background (The Jackson Laboratory), creating a new strain named *Sle16.Ldlr*^−/−^. From 10 weeks of age, experimental groups of female mice received either a high-fat or low-fat semisynthetic reference diet (Arieblok), whose compositions were previously reported (17). Animal care and procedures were conducted according to institutional guidelines.

### Atherosclerotic lesion analysis

After 12 weeks of the high-fat or low-fat diet, mice were killed by CO_2_ inhalation and then sequentially perfused, via the left ventricle, with Krebs-Hensleitt buffer, 2% formalin, and Sudan IV solution (17). The hearts and aortae were microdissected, and the aortic root was cryosectioned at 100-μm intervals and stained with oil red O. The aortae were cut open longitudinally, destained briefly in 80% ethanol, and photographed with an Olympus DP50 camera.

Quantification of atherosclerotic plaques on cross-sectional images of the aortic root and on whole aorta horizontal (en face) images was performed using ImagePro software (Media Cybernetics). Aortic root cryosections were stained using standard immunohistochemistry to identify the following cell types: macrophages (with MOMA-2 rat monoclonal antibodies; Serotec), vascular smooth muscle cells (VSMCs) (with alkaline phosphatase [AP]–conjugated anti–α-smooth muscle actin antibodies; Sigma-Aldrich), and T cells (with goat anti-mouse CD3ε antibodies; Santa Cruz Biotechnology). Apoptotic cells were detected using TUNEL staining (Roche). Randomized slides were quantified in a blinded manner, and assessed for the number of TUNEL-positive cells displaying morphologic criteria for apoptosis, including cell shrinkage, nuclear condensation, or fragmentation. Results from immunofluorescence analyses of the arterial lesions to detect C3 (with fluorescein isothiocyanate [FITC]–conjugated goat anti-mouse C3 antibodies; MP Biomedicals) and IgG (with FITC-conjugated goat anti-mouse IgG antibodies; Sigma-Aldrich) were quantified using ImagePro. Arterial lesion C3d was quantified as previously described (18).

### Measurement of serum lipids

The levels of total cholesterol and triglycerides in the serum of mice were measured using colorimetric enzymatic assays (Infinity; Alpha Laboratories).

### Measurement of anti–oxidized LDL (anti–ox-LDL) antibodies and autoantibodies

Human LDL (density 1.019–1.063 gm/ml) was obtained from the plasma of healthy human donors after a period of overnight fasting. The human LDL was isolated by differential density ultracentrifugation, and then oxidized with freshly synthesized malondialdehyde (MDA), to generate MDA-LDL. Anti–MDA-LDL antibodies were measured by enzyme-linked immunosorbent assay (ELISA), as previously described (17, 19). In addition, serum levels of anti–single-stranded DNA (anti-ssDNA), anti–double-stranded DNA (anti-dsDNA), antichromatin, antihistone, and anticardiolipin antibodies were assayed by ELISA (17). All ELISA results were expressed in arbitrary ELISA units, calculated relative to a reference standard from pooled MRL/*lpr* sera.

### Serum C3 ELISA

Microtiter plates were coated with a goat anti-mouse C3 antibody (Calbiochem) in 0.1*M* NaHCO_3_, and then blocked with an assay diluent of 2% phosphate buffered saline–bovine serum albumin. A biotinylated version of the capture antibody was used for detection, with the addition of AP-conjugated streptavidin. Quantification of serum C3 in the mice was achieved by reference to an acute-phase serum of known mouse C3 concentration (Calbiochem).

### Renal assessment

After the mice were killed, the kidneys were fixed in Bouin's solution, paraffin embedded, stained with periodic acid–Schiff, and scored for the presence of glomerulonephritis. Immunofluorescence staining for IgG and C3 in snap-frozen sections of the kidneys was quantified as previously described (16). Urinalysis dipstick (Haema-combistix; Bayer) was used to screen for proteinuria and hematuria. Serum urea was measured using a urea/ammonia ultraviolet method kit (Boehringer Mannheim/R-Biopharm) modified for use with mouse sera.

### Tail cuff blood pressure

Blood pressure (BP) was measured using noninvasive BP monitoring equipment for the mouse tail cuff (Model 229; IITC Life Science Instruments). Mice were acclimatized to the system prior to undergoing BP measurements. Five separate measurements of the systolic and diastolic BP were made per mouse over a 15-day period.

### Flow cytometry analysis of splenocytes

Flow cytometry was used to assess the mouse splenocytes, performed using a FACSCalibur flow cytometer (BD Biosciences), with results analyzed using FlowJo software (Tree Star). Since the flow cytometry data were distributed normally, two-way analysis of variance was used to analyze the effects of 2 factors simultaneously, with post hoc analysis using Student's *t*-tests with Bonferroni correction. Data were analyzed using SPSS software, version 16.0.

### Statistical analysis

Results were analyzed using the Prism program (version 3.0; GraphPad Software). The Mann-Whitney test was used to compare 2 groups, while the Kruskal-Wallis test with Dunn's post hoc test was used to compare 3 or more groups. Probability values were considered significant at *P* values less than 0.05.

## RESULTS

### Association of accelerated atherosclerosis with the *Sle16* locus

To examine the effect of the *Sle16* locus on atherosclerosis, we compared the extent of atherosclerosis in *Sle16.Ldlr*^−/−^ mice and *Ldlr*^−/−^ mice fed either a low-fat diet or a high-fat diet for 12 weeks. Low-fat diet–fed *Ldlr*^−/−^ mice typically develop mildly elevated levels of serum cholesterol and small, early-stage atherosclerotic lesions, whereas high-fat feeding causes massive elevations in the levels of cholesterol and larger, more advanced atherosclerotic plaques (20, 21).

**Figure 1:**
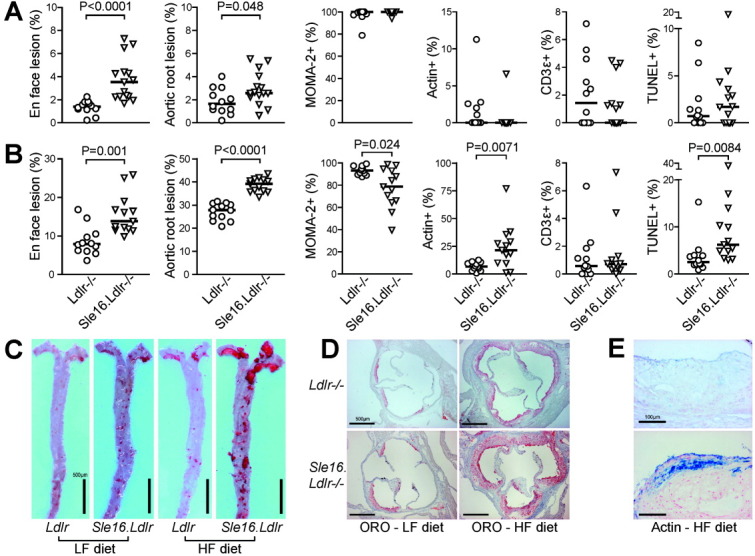
Accelerated atherosclerosis in *Sle16.Ldlr*^−/−^ mice compared to *Ldlr*^−/−^ control mice. A and B, In 22-week-old *Ldlr*^−/−^ and *Sle16.Ldlr*^−/−^ mice receiving either a low-fat (LF) (A) or high-fat (HF) (B) diet, the area of atherosclerotic lesions was quantified on horizontal (en face) images of the whole aorta and in cross-sectional images of the aortic root. In addition, lesional cell counts, expressed as the percentage of lesional cells with positively staining nuclei, were performed for macrophages (using MOMA-2 antibodies), vascular smooth muscle cells (VSMCs) (using anti–α-smooth muscle actin), T cells (using anti-CD3ε antibodies), and apoptotic cells (using TUNEL staining). Groups were compared by Mann-Whitney test. Symbols represent individual samples, and bars show the median. C and D, Representative Sudan IV–stained, en face images of the whole aorta (C) and photomicrographs of oil red O (ORO) staining of lesions in the aortic root (D) are shown. E, Results of staining for VSMCs with anti–α-smooth muscle actin antibodies (blue), are shown in representative high-power images from a high-fat–fed *Sle16.Ldlr*^−/−^ mouse (bottom) compared to an *Ldlr*^−/−^ control mouse (top), demonstrating fibrous cap formation in high-fat–fed *Sle16.Ldlr*^−/−^ mice.

Among mice receiving the low-fat diet, the introgression of the *Sle16* locus resulted in a 2.5-fold increase in the area of atherosclerotic lesions, as visualized on en face images of the whole aorta in *Sle16.Ldlr*^−/−^ mice (median lesional area 3.53%, range 1.69–7.29%; n = 15) compared to *Ldlr*^−/−^ control mice (median lesional area 1.40%, range 0.24–2.26%; n = 12) (*P* < 0.0001) (Figures 1A and C). At the aortic root, a 1.6-fold increase in lesional area was detected in *Sle16.Ldlr*^−/−^ mice compared to *Ldlr*^−/−^ mice (median lesional area 2.57%, range 0.64–5.54% in *Sle16.Ldlr*^−/−^ mice [n = 15] versus 1.64%, range 0.21–4.02% in *Ldlr*^−/−^ mice [n = 12]; *P* = 0.048) (Figures 1A and D).

Among mice receiving the high-fat diet, *Sle16.Ldlr*^−/−^ mice, compared to *Ldlr*^−/−^ control mice, showed a substantial increase in lesional area on whole aorta en face images (median lesional area 13.8%, range 9.78–25.9% in *Sle16.Ldlr*^−/−^ mice [n = 13] versus 7.85%, range 3.60–16.8% in *Ldlr*^−/−^ mice [n = 12]; *P* = 0.001) and a substantial increase in the fraction of aortic root lesional area (median 39.3%, range 33.5–43.6% in *Sle16.Ldlr*^−/−^ mice [n = 13] versus 27.8%, range 20.8–31.6% in *Ldlr*^−/−^ mice [n = 12]; *P* < 0.0001) (Figures 1B–D). There was no difference in body weight, serum cholesterol levels, or triglyceride levels between the 2 groups on either diet (details available from the corresponding author upon request). Atherosclerosis was not quantified in mice on the C57BL/6 background, since these lesions would have been too small to quantify reliably.

Histologic analysis of the lesion composition in the high-fat diet–fed mice revealed greater complexity in the *Sle16.Ldlr*^−/−^ mice compared to the *Ldlr*^−/−^ control mice, as shown by the increased percentage of VSMCs (*P* = 0.0071) and early fibrous cap formation in the *Sle16.Ldlr*^−/−^ mice (Figures 1B and E). This change in VSMC expression was accompanied by a reciprocal fall in proportional macrophage content (*P* = 0.024) (Figure 1B). In mice receiving either the low-fat or high-fat diet, no alteration in the percentage of lesional CD3ε-positive T cells was detected (Figures 1A and B). Overall, these data suggest that the high-fat diet on a lupus-prone background not only can induce a quantitative increase in atherosclerotic plaque burden, but also a qualitative change in plaque composition to more advanced lesions.

### Increased apoptosis in the arterial lesions of *Sle16.Ldlr*^−/−^ mice

Defective clearance of apoptotic cells is thought to play a role in the pathogenesis of SLE and has been proposed as one of the underlying mechanisms for the accelerated atherosclerosis in SLE (4). Aortic root sections from mice on either diet were TUNEL stained to quantify apoptosis in the lesions. Similar to the pattern observed for the plaque cellular compositions, no differences in apoptosis were observed in mice receiving the low-fat diet (Figure 1A), whereas in mice receiving the high-fat diet, a significant increase in the percentage of TUNEL-positive lesional cells was observed in the *Sle16.Ldlr*^−/−^ mice compared to the *Ldlr*^−/−^ control mice (median 6.22%, range 2.95–31.3% in *Sle16.Ldlr*^−/−^ mice [n = 13] versus 2.54%, range 0.86–15.3% in *Ldlr*^−/−^ control mice [n = 12]; *P* = 0.0084) (Figure 1B). This suggests that disposal of apoptotic cells within lesions may become impaired in larger plaques that are generated in response to high-fat feeding, although it is also possible that more apoptosis is occurring in larger plaques due to their more advanced nature.

### Evidence of hyperactive immune system in *Sle16.Ldlr*^−/−^ mice

**Figure 2:**
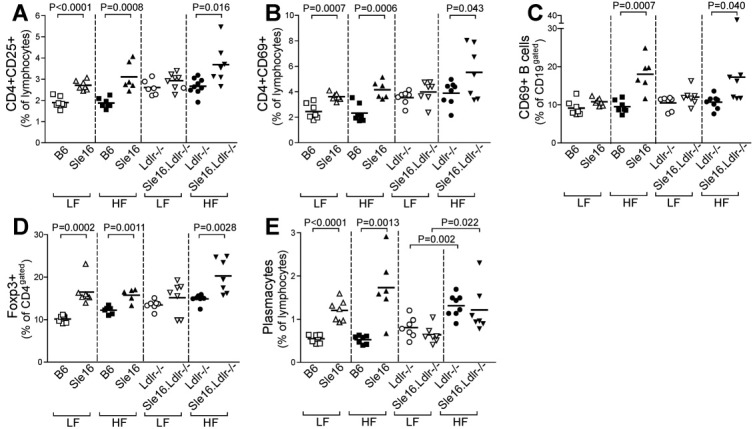
Synergistic enhancement of autoimmune activation of B and T lymphocytes by hyperlipidemia. The spleens of 22-week-old C57BL/6 (B6), *Sle16*, *Ldlr*^−/−^, and *Sle16.Ldlr*^−/−^ mice in either the low-fat (LF) or high-fat (HF) diet group were assessed for distribution of lymphocyte populations. Percentages of activated T cells, defined as either CD4+CD25+ (A) or CD4+CD69+ (B) T cells, activated B cells, defined as CD19+CD69+ B cells (C), Treg cells, defined as gated CD4+Foxp3+ cells (D), and plasmacytes, defined as CD138+ cells (E), were determined by flow cytometry. Symbols represent individual samples, and bars show the mean. Data were analyzed using two-way (*Sle16* × diet) analysis of variance. *P* values were determined by Student's *t*-test with post hoc analysis.

To investigate the cellular immune interactions of atherosclerosis and lupus, we performed flow cytometry analysis of splenocytes from C57BL/6, *Sle16*, *Ldlr*^−/−^, and *Sle16.Ldlr*^−/−^ mice in both diet groups. The most substantial changes were seen in markers of lymphocyte activation. High-fat–fed *Sle16.Ldlr*^−/−^ mice exhibited an overactive phenotype, characterized by an increased percentage of both CD25+ (*P* = 0.016) and CD69+ (*P* = 0.043) CD4+ T cells when compared to *Ldlr*^−/−^ mice (Figures 2A and B), as well as an enhanced percentage of CD69+ B cells (*P* = 0.040) (Figure 2C). Interestingly, the increased activation of B and T cells was accompanied by an expansion of the CD4+Foxp3+ Treg cell subset (*P* = 0.003) (Figure 2D), possibly occurring as a result of compensatory mechanisms.

Synergy between the genetic strains was apparent, with a hierarchical increase in the percentage of the 3 T cell subsets in *Ldlr*^−/−^ mice compared to C57BL/6 mice, and a further increase in *Sle16.Ldlr*^−/−^ mice, particularly on the high-fat diet. The *Sle16* locus is known to increase the numbers of activated CD69+CD4+ T cells and Treg cells (22). However, the differences between *Sle16*.*Ldlr*^−/−^ and *Ldlr*^−/−^ mice were predominantly observed in the high-fat diet–fed groups, suggesting that an environment of hyperlipidemia may exacerbate the activation state typical of an autoimmune background. Consistent with this, we noticed an increased percentage of CD138+ plasmacytes in the high-fat diet–fed cohorts compared to the low-fat diet–fed groups (*P* = 0.002 in high-fat–fed versus low-fat–fed *Ldlr*^−/−^ mice; *P* = 0.022 in in high-fat–fed versus low-fat–fed *Sle16.Ldlr*^−/−^ mice) (Figure 2E).

### Serologic consequences of the coexistence of hyperlipidemia and autoimmunity

#### Effects of the Sle16 locus on anti–ox-LDL antibody production

**Figure 3:**
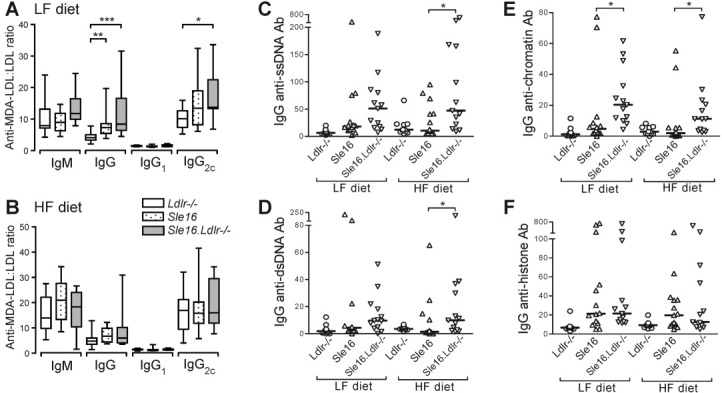
Effects of hyperlipidemia on serum autoantibody levels. A and B, Serum levels of anti–malondialdehyde–low-density lipoprotein (anti–MDA-LDL) antibodies were determined by enzyme-linked immunosorbent assay (ELISA) in 22-week-old *Ldlr*^−/−^, *Sle16*, and *Sle16.Ldlr*^−/−^ mice receiving either the low-fat (LF) (A) or high-fat (HF) (B) diet. ELISAs were performed at a serum dilution of 1:80 for IgM, 1:20 for IgG, and 1:5 for IgG1 and IgG2c. Antibody titers were determined as the ratio of anti–MDA-LDL antibodies to anti–native LDL antibodies (at an absorbance of 405 nm), with results presented as box plots, where the boxes represent the 25th to 75th percentiles, the lines within the boxes represent the median, and the lines outside the boxes represent the 10th and 90th percentiles. C–F, Serum levels of IgG anti–single stranded DNA (anti-ssDNA) (C), anti–double-stranded DNA (anti-dsDNA) (D), antichromatin (E), and antihistone (F) antibodies (Ab) were determined by ELISA in 22-week-old *Ldlr*^−/−^, *Sle16*, and *Sle16.Ldlr*^−/−^ mice in either diet group. Results are expressed as arbitrary ELISA units, relative to a reference standard of pooled MRL/*lpr* sera (assigned a value of 100 AEU). Symbols represent individual samples, and bars show the median. * = *P* < 0.05; ** = *P* < 0.01; *** = *P* < 0.001, by Kruskal-Wallis test with Dunn's post hoc test.

We measured serum anti–ox-LDL antibody levels using MDA-LDL. In mice receiving the low-fat diet, a marked increase in IgG anti–MDA-LDL antibodies was observed in *Sle16.Ldlr*^−/−^ mice compared to *Ldlr*^−/−^ control mice (*P* < 0.001), with a trend toward an increase in IgM anti–MDA-LDL antibodies (Figure 3A).

Hypercholesterolemia has been associated with a Th1-to-Th2 shift (19), and therefore IgG subclasses were analyzed. The IgG1 titer was not different, whereas the IgG2c titer was significantly increased in *Sle16.Ldlr*^−/−^ mice compared to *Ldlr*^−/−^ control mice (Figure 3A). In the high-fat diet group, however, no significant differences in the levels of anti–MDA-LDL antibodies of any isotype were detected between *Ldlr*^−/−^ and *Sle16.Ldlr*^−/−^ mice (Figure 3B), which could be mainly attributed to the increased levels of anti–ox-LDL antibodies in *Ldlr*^−/−^ mice in response to the elevated hyperlipidemia driven by the high-fat diet, as has been previously reported (23).

#### Effects of hyperlipidemia on lupus autoantibody production

To examine whether hyperlipidemia could alter the autoimmune serologic profile driven by the *Sle* locus, we assessed a wide range of lupus autoantibodies. As expected, *Ldlr*^−/−^ mice showed negligible levels of these autoantibodies, and thus the most informative comparison was between the *Sle16.Ldlr*^−/−^ mice and the lupus-prone *Sle16*-congenic mice (Figures 3C–F). On the high-fat diet, *Sle16.Ldlr*^−/−^ mice developed significantly higher titers of anti-ssDNA, anti-dsDNA, and antichromatin antibodies compared to the *Sle16* animals (Figures 3C–E), but antihistone antibody titers were not different (Figure 3F). In mice receiving the low-fat diet, a similar trend toward statistically significant differences was seen for all 4 autoantibodies, but only antichromatin antibody levels were significantly different between the 2 strains. No significant differences in IgG or IgM anticardiolipin antibody levels were observed in mice receiving either the low-fat or the high-fat diet.

#### Effects of coexistence of hyperlipidemia and autoimmunity on targeted organs

**Figure 4:**
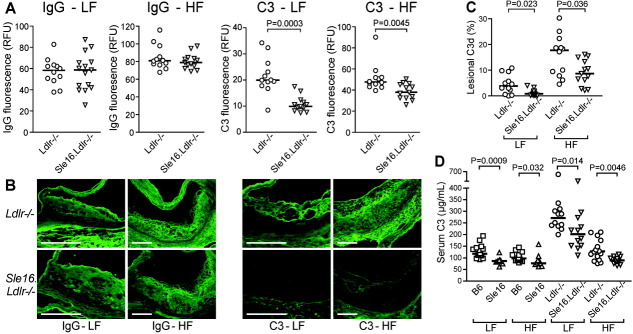
Lack of effect of the *Sle16* locus on immune complex or complement deposition in vascular lesions. A, The density of IgG and C3 deposition in the lesional aortic root in groups of *Ldlr*^−/−^ and *Sle16.Ldlr*^−/−^ mice receiving either the low-fat (LF) or high-fat (HF) diet was measured as the mean fluorescence intensity per pixel, with results expressed in relative fluorescence units (RFU). B, Representative photomicrographs show lesional IgG and C3 deposition in the aortic root of low-fat– and high-fat–fed *Ldlr*^−/−^ and *Sle16.Ldlr*^−/−^ mice. Bars = 100 μm (original magnification × 20 in the low-fat groups; × 10 in the high-fat groups). C, C3d deposition in the lesional aortic root was measured by immunohistochemistry in low-fat– and high-fat–fed *Ldlr*^−/−^ and *Sle16.Ldlr*^−/−^ mice, with results expressed as the percentage of lesional area showing C3d deposition. D, Serum C3 levels were measured by enzyme-linked immunosorbent assay in low-fat– and high-fat–fed *Ldlr*^−/−^ and *Sle16.Ldlr*^−/−^ mice. Symbols represent individual samples, and bars show the median. *P* values were determined by Mann-Whitney test.

The *Sle16* locus does not induce accelerated atherosclerosis by enhancing IC or C3 deposition in the arterial lesions. An overall increase in the levels of IgG and IgM anti–ox-LDL antibodies was observed in *Sle16.Ldlr*^−/−^ mice compared to *Ldlr*^−/−^ control mice. These antibodies can be detected as molecules bound to modified lipids in atherosclerotic plaques (24), and therefore one might expect to find increased levels of IgG deposition in the atherosclerotic lesions of *Sle16.Ldlr*^−/−^ mice, as well as more pronounced local complement activation. The deposition of IgG and C3 in adjacent aortic root cryosections was thus quantified. There was no difference in the density of lesional IgG deposition in *Sle16.Ldlr*^−/−^ mice compared to *Ldlr*^−/−^ control mice in either diet group (Figures 4A and B). Surprisingly, C3 deposition was significantly lower in *Sle16.Ldlr*^−/−^ mice compared to *Ldlr*^−/−^ mice (*P* = 0.0003 for the low-fat diet groups and *P* = 0.0045 for the high-fat diet groups) (Figures 4A and B), and this was confirmed by quantification of lesional C3d deposition (Figure 4C). These findings prompted us to measure the levels of C3 in the plasma.

Notably, *Sle16.Ldlr*^−/−^ mice had significantly lower circulating C3 levels compared to diet-matched *Ldlr*^−/−^ mice, with the difference being more pronounced in the high-fat environment (*P* = 0.014 for the low-fat diet groups and *P* = 0.0046 for the high-fat diet groups). Therefore, the reduction in lesional C3 deposition in *Sle16.Ldlr*^−/−^ mice appeared to mirror the fall in plasma C3 levels. Since lesional C3d deposition was lower in *Sle16.Ldlr*^−/−^ mice, this suggests that lower serum C3 levels are not due to in situ complement activation within atherosclerotic plaques (although complement activation may also be occurring elsewhere). It also implies that intralesion complement activation is not the cause of the accelerated atherogenesis in the *Sle16.Ldlr*^−/−^ mouse autoimmune model.

Circulating C3 levels were also lower in *Sle16* mice compared to C57BL/6 control mice, confirming that the autoimmune *Sle16* locus has a consistent lowering effect on serum C3 (*P* = 0.0009 for the low-fat diet groups and *P* = 0.032 for the high-fat diet groups), which is most likely related to IC-mediated complement consumption. Furthermore, we observed higher levels of serum C3 in low-fat diet–fed *Ldlr*^−/−^ mice compared to C57BL/6 control mice (Figure 4D). The cause for the rise in C3 in low-fat–fed *Ldlr*^−/−^ mice is unknown and previously unreported, but we hypothesize that it may be due to an acute-phase response, as short-term hyperlipidemia is known to elevate serum C3 in *ApoE*^−/−^ mice (25).

### Enhancement of IC-mediated renal inflammation by hyperlipidemia via amplification of complement activation

**Figure 5:**
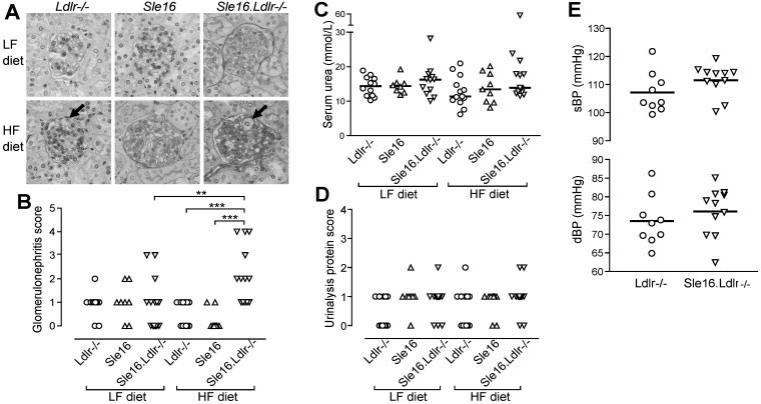
Promotion of renal inflammation by high-fat feeding in *Sle16.Ldlr*^−/−^ mice. A, Representative photomicrographs show periodic acid–Schiff–stained kidney sections from 22-week-old *Ldlr*^−/−^, *Sle16*, and *Sle16.Ldlr*^−/−^ mice in either the low-fat (LF) or high-fat (HF) diet group, demonstrating increased glomerular hypercellularity in the high-fat–fed *Sle16.Ldlr*^−/−^ mice. Numerous foam cells were noted in glomeruli of all *Ldlr*-deficient strains after high-fat feeding (arrows). B, Glomerulonephritis was scored in each group using a renal histologic severity scale of 0–5 (see ref.^16^). * = *P* < 0.01; ** = *P* < 0.001, by Kruskal-Wallis test with Dunn's post hoc test. C and D, Serum urea levels (C) and proteinuria (D) were determined in the groups of mice at 22 weeks old. The protein score on urinalysis was graded as follows: grade 0 = <0.30 gm/liter, grade 1 = ≥0.30 gm/liter, grade 2 = ≥1 gm/liter, grade 3 = ≥3 gm/liter, grade 4 = ≥20 gm/liter. E, Tail cuff systolic blood pressure (sBP) and diastolic blood pressure (dBP) were determined in 52-week-old high-fat–fed *Ldlr*^−/−^ and *Sle16.Ldlr*^−/−^ mice. Results are expressed as the mean of 5 measurements obtained twice weekly. Symbols represent individual results, and bars show the median.

Consistent with previous findings (16), *Sle16* mice receiving the low-fat diet developed changes related to the development of mild, proliferative glomerulonephritis, and similar histologic changes were noted in low-fat diet–fed *Sle16.Ldlr*^−/−^ mice (Figures 5A and B). However, in *Sle16.Ldlr*^−/−^ mice, the high-fat diet led to a striking increase in the severity of renal inflammation, both in comparison to *Sle16* mice (*P* < 0.001) and in comparison to low-fat diet–fed *Sle16.Ldlr*^−/−^ mice (*P* < 0.01). There was no histologic evidence of tubulointerstitial damage in any cohort.

We found no significant differences in the serum urea levels (Figure 5C). Moreover, none of the cohorts developed significant proteinuria (Figure 5D). No differences in blood pressure were detected between *Ldlr*^−/−^ and *Sle16.Ldlr*^−/−^ mice (Figure 5E). Therefore, the advanced atherosclerosis in the lupus-prone animals was not due to declining renal function and/or hypertension.

**Figure 6:**
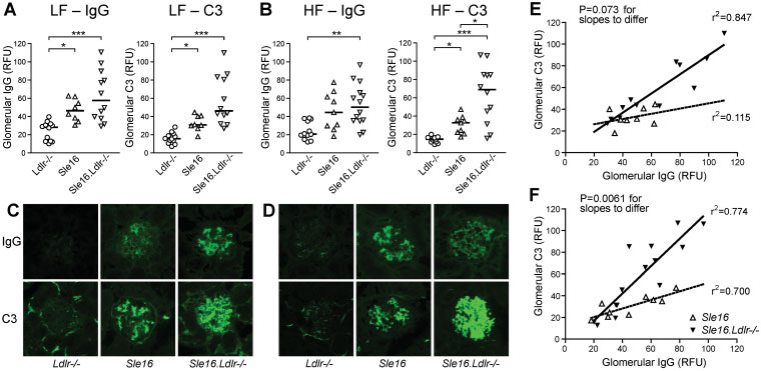
Association of hyperlipidemia with amplified glomerular C3 deposition, but not with increased IgG deposition, in *Sle16.Ldlr*^−/−^ mice. A and B, Glomerular IgG and C3 depostion was quantified in *Ldlr*^−/−^, *Sle16*, and *Sle16.Ldlr*^−/−^ mice receiving either the low-fat (LF) (A) or high-fat (HF) (B) diet. Results are expressed as relative fluorescence units (RFU). Symbols represent individual samples, and bars show the median. * = *P* < 0.05; ** = *P* < 0.01; *** = *P* < 0.001, by Kruskal-Wallis test with Dunn's post hoc test. C and D, Representative images of glomeruli show immunofluorescence staining for IgG and C3 in mice receiving either the low-fat (C) or high-fat (D) diet. Original magnification × 40. E and F, Correlation plots show linear relationships between glomerular IgG deposition and glomerular C3 deposition in the *Sle16* and *Sle16.Ldlr*^−/−^ mice receiving either the low-fat (E) or high-fat (F) diet. Correlation (r^2^) values are Pearson's correlation coefficients. *P* values for discordance in the gradients of the lines of best fit were calculated by analysis of covariance.

We then quantified the amount of IgG and C3 deposited in the kidneys. *Ldlr*^−/−^ control mice showed minimal levels of glomerular IgG and C3. IgG deposition was significantly increased in *Sle16.Ldlr*^−/−^ mice compared to *Ldlr*^−/−^ mice on either diet (*P* < 0.001 for the low-fat diet groups and *P* < 0.01 for the high-fat diet groups). Notably, we found no significant differences in IgG deposition between *Sle16.Ldlr*^−/−^ and *Sle16* mice on either diet (Figures 6A–D). In contrast, with regard to C3 deposition, a hierarchy was apparent, in that *Sle16* mice showed higher levels of C3 than did *Ldlr*^−/−^ mice on either diet (both *P* < 0.05), but significantly less C3 deposition when compared with the *Sle16.Ldlr*^−/−^ mice on the high-fat diet (*P* < 0.05) (Figures 6B and D), with a trend toward a decrease in serum C3 in the low-fat diet group of *Sle16.Ldlr*^−/−^ mice (Figures 6A and C). In addition, whereas the pattern of deposition of both C3 and IgG was mesangial in *Sle16* mice, capillary wall deposition of C3 was evident in the high-fat diet–fed *Sle16.Ldlr*^−/−^ mice (Figure 6D).

The finding that *Sle16.Ldlr*^−/−^ mice had enhanced renal C3 deposition that was above and beyond that seen in *Sle16* mice was at odds with the failure to demonstrate an increase in IgG staining. This raised the possibility that there could be discordance between the level of glomerular C3 deposition and the level of glomerular IgG deposition in these cohorts. To examine the relationship between these 2 parameters within the kidney, we generated correlation plots, as shown in Figures 6E and F. The correlation plots showed a clearly linear relationship between glomerular IgG levels and glomerular C3 levels in both low-fat diet– and high-fat diet–fed *Sle16.Ldlr*^−/−^ mice (r^2^ = 0.847, *P* < 0.0001 for the low-fat diet group; r^2^ = 0.774, *P* < 0.0001 for the high-fat diet group), and in the high-fat diet–fed *Sle16* mice (r^2^ = 0.700, *P* = 0.0049); however, this relationship was not evident in low-fat diet–fed *Sle16* mice (r^2^ = 0.115, *P* = 0.41).

Interestingly, a significant discordance was noted in the gradients of the 2 lines of best fit between high-fat–fed *Sle16.Ldlr*^−/−^ mice and high-fat–fed *Sle16* mice (*P* = 0.0061 by analysis of covariance) (Figure 6F), indicating that, for a given level of glomerular IgG, a higher amount of C3 was deposited within the kidneys of the *Sle16.Ldlr*^−/−^ mice compared to the *Sle16* mice. Although the difference in the ratio of C3:IgG deposition reached strong statistical significance when comparing *Sle16.Ldlr*^−/−^ mice and *Sle16* mice receiving the high-fat diet, it showed only a statistical trend (*P* = 0.073) in mice receiving the low-fat diet, with a less steep gradient. These findings suggest that hyperlipidemia enhances the IC-mediated renal damage by amplifying complement deposition.

## DISCUSSION

The cellular mechanisms underlying the marked increased risk of atherosclerosis and its complications in patients with SLE remain poorly understood and cannot be explained by traditional risk factors (26). In the present study, we crossed a lupus-prone strain, *Sle16-* congenic mice (16), with atherosclerosis-prone *Ldlr*^−/−^ mice and showed that the combination of SLE and hyperlipidemia led to the enhancement of both atherosclerosis and renal inflammation. More importantly, by comparing cohorts of mice on different diets, our data provide, for the first time, evidence that distinct pathways promote the accelerated tissue injury in the affected organs.

In the kidney, hyperlipidemia enhanced IC-mediated renal damage by amplifying local complement deposition, a mechanism that did not appear to operate within the arterial walls. On the contrary, circulating complement consumption, rather than in situ activation, appeared to be linked to accelerated atherogenesis, an observation that is consistent with the proposed protective role of complement in debris disposal within atherosclerotic lesions (27). The results from our animal model, in agreement with the clinical observations in humans and distinct from the previous observations in murine studies (4–8), highlights the detrimental effects of hyperlipidemia not only on atherosclerotic plaque formation, but also on IC-mediated renal inflammation.

Most previous murine studies have used *lpr* or *gld* mice, which have defects in Fas and the Fas ligand, respectively (4, 6–8), leading to defective apoptosis within lymphoid tissue, severely impaired T cell differentiation, and massive hepatosplenomegaly and lymphadenopathy in mice, and the lymphoma-like Canale-Smith syndrome in humans (28). Therefore, these models do not closely resemble human SLE. An alternative model was the triple-congenic chimera *Sle1.2.3→Ldlr*^−/−^, proposed by Stanic et al (5), which has similarities to our model, since *Sle1* overlaps with *Sle16* (16, 29). However, the combination of 3 SLE loci (*Sle1*, *Sle2*, and *Sle3)* led to severe autoimmune disease, with evidence of renal impairment and death, that might have contributed to the accelerated atherosclerosis. These confounding effects have been overcome in our model, and each phenotype was compared against the most appropriate control strain, thus providing more informative comparisons.

In both diet groups, *Sle16.Ldlr*^−/−^ mice showed strikingly more extensive atherosclerotic lesions compared to *Ldlr*^−/−^ control mice. Not only was the total burden of atherosclerosis greater, but in high-fat diet–fed *Sle16.Ldlr*^−/−^ mice, the lesions were more complex, with increased infiltration of VSMCs. Despite the effects of the *Sle16* locus on anti–ox-LDL and lupus autoantibody titers, *Sle16.Ldlr*^−/−^ mice displayed similar IgG deposition in the aortic root plaques when compared to *Ldlr*^−/−^ control mice. This contrasted with the marked decrease in complement C3 deposition within atherosclerotic plaques in mice receiving either diet. This unexpected result clearly demonstrates that, contrary to the common belief, lupus may not lead to accelerated atherosclerosis by driving increased IC deposition within vascular lesions, and other immunologic mechanisms may be more critical. This is in keeping with the findings from human studies, which have failed to show an association between lupus autoantibody titers and vascular events (2, 30).

Deficiency of C3 on the *Ldlr*^−/−^ mouse background has been shown to result in larger atherosclerotic lesions, with increased lipid deposition and impaired lesion development beyond the foam cell stage (31), while the classical and lectin complement pathways protect against atherosclerosis (10, 32). Thus, lower levels of plaque C3, most likely due to circulating complement consumption, may be an important underlying mechanism for the increased plaque size in *Sle16.Ldlr*^−/−^ mice. C3 is important for uptake of dying cells (33), and therefore the reduction in C3 within plaques could also explain the increased levels of TUNEL-positive apoptotic cells observed in *Sle16*.*Ldlr*^−/−^ mouse lesions.

Increased numbers of apoptotic cells within atherosclerotic lesions have been described in previous models (4, 6, 7). However, none of these earlier studies directly linked apoptosis in the arterial lesions with reduced levels of complement, and several studies are confounded by the use of *ApoE*^−/−^ mice, which have an intrinsic macrophage defect in phagocytosis of apoptotic cells (34). Defective clearance of apoptotic cells through deficiency of complement components has been linked to autoimmunity, indicating that impaired waste disposal mechanisms may operate synergistically in both conditions (9).

The reduction in lesional C3 reflected the fall in serum C3 levels in *Sle16.Ldlr*^−/−^ mice compared to *Ldlr*^−/−^ control mice in both diet groups. This observation has 2 important implications. First, plaque complement levels are directly influenced by circulating levels, possibly by diffusion of C3 from the circulation through the vessel wall. Second, the hyperlipidemic environment affects autoantibody production and subsequent IC-mediated complement consumption. Consistent with the latter notion, in the *Sle16.Ldlr*^−/−^ mice, we found a trend toward increased levels of autoantibodies compared to those in *Sle16* autoimmune mice. Unsurprisingly, in the spleens from high-fat–fed *Sle16.Ldlr*^−/−^ mice, we detected significantly increased percentages of activated B and T cells when compared to *Ldlr*^−/−^ mice. Hyperlipidemia itself appeared to have a strong, independent proliferative effect on plasmacytes. These results again suggest that synergy between the 2 conditions may occur, with hyperlipidemia amplifying the autoimmune phenotype.

Although we cannot rule out an effect of *Sle16* on hepatic synthesis of C3, we postulate that the reduction in circulating C3 was most probably the result of IC deposition within the affected organs, particularly the kidney. In this regard, it was essential to compare *Sle16*.*Ldlr*^−/−^ mice with lupus-prone *Sle16* mice, which develop very mild glomerulonephritis (16). The combination of the *Sle16* locus with LDL receptor deficiency led to the development of glomerulonephritis in *Sle16.Ldlr*^−/−^ mice, but only in the context of the high-fat diet. These changes in glomerular inflammation did not cause increased proteinuria, renal failure, or hypertension. Both autoimmune strains, *Sle16* and *Sle16.Ldlr*^−/−^, displayed an equal increase in glomerular IgG deposition when compared to *Ldlr*^−/−^ control mice. However, *Sle16.Ldlr*^−/−^ mice showed higher levels of glomerular C3 deposition than did either the *Sle16* or the *Ldlr*^−/−^ animals, with the highest levels of C3 seen on the high-fat diet. While C3 levels were linearly correlated with IgG deposition, the increased gradient of C3:IgG deposition in high-fat–fed *Sle16.Ldlr*^−/−^ mice suggests that the level of hyperlipidemia affected the amount of C3 deposition and renal damage.

High-fat diets in nonatherosclerotic (NZB × NZW)F_1_ mice with lupus have also been shown to exacerbate glomerulonephritis and reduce survival (35–37). A likely explanation for our findings is that the proatherogenic lipid profile of mice with the *Ldlr*^−/−^ background led to the deposition of ox-LDL and/or enzymatically modified LDL within glomeruli, causing complement activation. High-fat feeding was necessary to trigger renal inflammation; this could be attributed to a combination of enhanced activation of the alternative pathway (18, 38, 39) and B and T cell activation, as seen in the flow cytometry results. A possible alternative explanation for altered C3 deposition in atherosclerotic and glomerular lesions in *Sle16.Ldlr*^−/−^ mice is differential IgG subclass deposition. We believe this is unlikely, since the IgG subclass is not correlated with glomerular C3 deposition in *Sle16* mice (Botto M: unpublished data). The results of our study demonstrate that hyperlipidemia can interact with autoimmunity to prime the kidney to IC-mediated injury.

Previous studies examining the links between SLE and atherosclerosis have focused on 2 key areas: IC deposition, and defective clearance of apoptotic cells within vascular lesions. IC deposition is undoubtedly a fundamental aspect of lupus pathogenesis. However, the role of the antibody response to oxidatively modified lipoproteins in atherosclerosis remains controversial. IgG and IgM anti–ox-LDL antibodies can be detected in abundance within atherosclerotic lesions, and may be pathogenic (40, 41). Yet, there is compelling evidence that some anti–ox-LDL antibodies, in particular IgM, are strongly atheroprotective (17, 42). In the present study, the combination of SLE and atherosclerosis led to exacerbation of both conditions. However, the accelerated atherosclerosis was not driven by an exacerbation of IC deposition within plaques, and the actions of complement appear to be distinct, in that they appear to be pathogenic in the glomerulus, but protective in atherosclerotic lesions.

In summary, our study findings show that lipid metabolism, the complement system, and autoimmunity are more intertwined than was previously appreciated. It provides additional strong support for a recommendation of aggressive treatment of hyperlipidemia in patients with SLE, in order to reduce both cardiovascular risk and complement-mediated renal damage. A major vindication of this proposal is provided in the findings from a recent clinical study, which demonstrated that serum total cholesterol levels >5.2 mmoles/liter at baseline were associated with renal deterioration/death in a cohort of 1,060 patients with SLE (43).

## AUTHOR CONTRIBUTIONS

All authors were involved in drafting the article or revising it critically for important intellectual content, and all authors approved the final version to be published. Dr. Botto had full access to all of the data in the study and takes responsibility for the integrity of the data and the accuracy of the data analysis.

**Study conception and design.** Lewis, Haskard, Botto.

**Acquisition of data.** Lewis, Malik, Fossati-Jimack, Carassiti, Cook, Botto.

**Analysis and interpretation of data.** Lewis, Haskard, Botto.
